# Urinary 6-sulfatoxymelatonin as a predictive biomarker for brain injury in very preterm infants

**DOI:** 10.1038/s41598-026-42005-0

**Published:** 2026-02-27

**Authors:** Yan Wang, Jincheng Zeng, Jinzhen Su, Yanyong Liang, Minxu Li

**Affiliations:** 1 Department of Neonatology, Dongguan Maternal and Child Health Care Hospital, Dongguan, Guangdong China; 2 Dongguan Key Laboratory of Medical Bioactive Molecular Developmental and Translational Research, Guangdong Provincial Key Laboratory of Medical Immunology and Molecular Diagnostics, Guangdong Medical University, Dongguan, Guangdong China

**Keywords:** 6-sulfatoxymelatonin, brain injury, very preterm infants, biomarker, melatonin, Biomarkers, Diseases, Medical research, Neurology, Neuroscience

## Abstract

Brain injury in preterm infants (BIPI) remains a significant clinical challenge with limited diagnostic biomarkers. This study aimed to investigate urinary 6-sulfatoxymelatonin (6-SMT) levels as a potential noninvasive biomarker for brain injury in very preterm infants. A prospective cohort study was conducted with 127 very preterm infants admitted to our hospital from January to December 2024. Infants were categorized into brain-injury and control group based on neuroimaging findings. Urinary 6-SMT concentrations were measured on postnatal days 1, 3, and 7 using ELISA. Clinical parameters and perinatal risk factors were evaluated. Receiver operating characteristic (ROC) curve analysis was used to assess diagnostic efficacy, and conditional logistic regression analysis was performed to evaluate the association between urinary 6-SMT levels and brain injury. Compared with the control group (*n* = 97), infants with brain injury (*n* = 30) exhibited significantly lower gestational age (GA: 29.36 vs. 30.43 weeks, *p* = 0.028) and birth weight (BW: 1.21 vs. 1.34 kg, *p* = 0.030), significantly lower rates of acidosis (10.0% vs. 12.4%, *p* = 0.023) and antenatal magnesium sulfate (MgSO₄) exposure (30.0% vs. 60.8%, *p* = 0.003), and significantly higher rates of early infection (46.7% vs. 21.6%, *p* = 0.007) and asphyxia (30.0% vs. 1.0%, *p* = 0.014). Crucially, urinary 6-SMT levels were markedly lower in the brain injury group on all measurement days (day 1: 558.51 vs. 813.86 pg/mL, *p* = 0.015; day 3: 722.62 vs. 938.48 pg/mL, *p* < 0.001; Day 7: 796.81 vs. 1034.48 pg/mL, *p* = 0.014). ROC analysis identified day 3 urinary 6-SMT (cut-off 762.46 pg/mL) as the best single marker (AUC = 0.714, sensitivity 73.2%, specificity 70.0%), while a combined model integrating levels from days 1, 3, and 7 achieved superior diagnostic performance (AUC = 0.764, sensitivity 78.4%, specificity 66.7%). Urinary 6-SMT levels progressively increased during the first postnatal week and correlated positively with GA and BW on days 1 and 3, but correlations weakened by day 7. After 1:1 matching on GA and BW, conditional logistic regression analysis confirmed a significant protective association between higher day 3 urinary 6-SMT levels and reduced brain injury risk (adjusted OR = 0.996, *p* = 0.004). Reduced urinary 6-SMT concentrations in very preterm neonates are significantly associated with brain injury. Serial urinary 6-SMT measurements, particularly when combined across multiple time points, demonstrate promising diagnostic potential as a noninvasive biomarker for BIPI. These findings suggest that melatonin deficiency may contribute to the pathophysiology of preterm brain injury and warrant further investigation for clinical translation.

**Supplementary Information:**

The online version contains supplementary material available at 10.1038/s41598-026-42005-0.

## Introduction

Preterm delivery remains a major public-health concern. In China, the preterm birth rate is 8%-10%, yielding approximately 1.2–1.5 million preterm infants annually; roughly 300,000 are born at < 32 weeks gestation. Advances in perinatal care have improved survival, yet 25%-50% of BIPI develop cognitive, behavioral or social deficits and 5%-10% acquire cerebral palsy^1,2^, placing a heavy burden on families and society. Early, objective and non-invasive markers of brain injury are therefore urgently needed.

Melatonin, a pineal-derived indole mainly synthesized from tryptophan, regulates circadian rhythm and exerts antioxidant, anti-inflammatory, anti-apoptotic and anti-excitotoxic effects^3,4^. In neonatal models, melatonin reduces oxidative damage in chronic lung disease, necrotizing enterocolitis, retinopathy of prematurity, sepsis and perinatal brain injury^5,6^. In animal models of BIPI, melatonin also attenuates motor dysfunction and white-matter loss^7,8^. However, clinical evidence linking endogenous melatonin status to brain injury in very preterm infants is scarce, and predictive thresholds have not been established.

As melatonin’s primary metabolite, 6-SMT can be noninvasively quantified in urine, providing an alternative to blood melatonin assessment^9,10^. The primary aim of our study was to investigate serial 6-SMT levels during the first postnatal week in very preterm infants and assess its diagnostic and pathophysiological relevance to brain injury. Building upon evidence suggesting melatonin’s neuroprotective role, we hypothesized that reduced urinary 6-SMT concentrations would be associated with brain injury in this vulnerable population. To test this, we measured urinary 6-SMT levels on days 1, 3, and 7 and compared profiles between preterm infants with and without brain injury. Furthermore, we aimed to identify diagnostic cut-off values and evaluate the predictive power of combining measurements across multiple time points.

## Methods

### Study population

All inborn infants with gestational age < 32 weeks born between January 1, 2024 and December 31, 2024 at Dongguan Maternal and Child Health Care Hospital were consecutively screened for eligibility (Fig. S1). Of 223 infants screened, 127 met the inclusion criteria and were prospectively enrolled. Based on imaging findings (ultrasound or magnetic resonance imaging), 30 were assigned to the brain injury group and 97 to the no brain injury group. Brain injuries covered white matter injuries and intraventricular hemorrhages. The study was approved by the Ethics Committee of Dongguan Maternal and Child Health Care Hospital, and informed consent was obtained from all legal guardians. The protocol followed the Declaration of Helsinki’s requirements.

### Inclusion and exclusion criteria

Inclusion criteria: (1) the infant underwent cranial magnetic resonance imaging; (2) the infant was born and admitted to our neonatal intensive care unit; and (3) the parents or legal guardians of the infant provided written informed consent.

Exclusion criteria: (1) a family history of known bleeding disorders, congenital neurological malformations, or other comorbidities such as genetic or metabolic disorders; (2) unwillingness of the parents or legal guardians to participate in the study; and (3) incomplete information, including cases where the infant was transferred to another hospital during treatment or where treatment was abandoned.

### Diagnostic criteria for brain injury in very preterm infants

Preterm infants underwent an initial cranial ultrasound within 3 days of birth, followed by weekly checkups until discharge. A cranial MRI was performed before discharge or at 40 weeks of corrected gestational age. Brain injury was defined as the presence of any grade of intraventricular hemorrhage and/or white-matter injury. IVH was graded by cranial ultrasound using the Papile classification^11^: grade 1, germinal-matrix hemorrhage without ventricular extension; grade 2, IVH with ≤ 50% ventricular filling without dilatation; grade 3, IVH with > 50% filling and acute ventricular dilatation; and grade 4, parenchymal hemorrhage or venous infarction. White-matter injury was defined as either cystic periventricular leukomalacia or persistent punctate white-matter lesions lasting > 7 days on MRI performed at term-equivalent age^12^. For severity stratification, mild brain injury was defined as Papile grades 1–2 or punctate persistent white-matter lesions, whereas severe brain injury was defined as Papile grades 3–4 or cystic periventricular leukomalacia.

### General information

Demographic and clinical variable data linked to clinical risk factors for brain injury in preterm infants were collected. The basic clinical characteristics include gender, gestational age, birth weight, delivery mode (vaginal or cesarean), acidosis (metabolic or respiratory), asphyxia (defined as any of the following: 1 or 5-min Apgar score ≤ 7, or umbilical-artery pH < 7.2), antenatal corticosteroids (ACS), antenatal magnesium sulfate, and early infection (defined as any culture-proven or clinically suspected infection occurring within 48 h of birth). All these are listed in Table 1.

### 6-SMT tests

On postnatal days 1, 3 and 7, 1 mL of urine was collected from each infant. Boys received a sterile adhesive pediatric urine bag (Hollister U-Bag) applied to the cleansed perineum; the bag was removed immediately after spontaneous voiding. Girls were first wiped with sterile water-soaked cotton balls, a single cotton ball was placed between the labia, and the same type of urine bag was applied; once saturated, the cotton ball was compressed with sterile forceps to express the sample. Any specimen contaminated with visible stool or blood was discarded. Urine samples were centrifuged at 1000 r/min for 20 min, and the supernatant was used for testing. Urinary 6-SMT levels were measured by ELISA using a kit (Jonlnbio Industrial Co., China).

### Statistics

Data analysis used SPSS 21.0 and R 4.3.1. Continuous data were tested for normality. Non-normally distributed data were expressed as median (inter-quartile range) and analyzed using the non-parametric test for between-group comparisons. Count data were presented as rates (%) and compared via chi-square tests. Pearson’s correlation analysis was used to assess associations. Receiver operating characteristic (ROC) curve analysis was used to determine the cut-off values of area under the curve (AUC) of urinary 6-SMT levels and identify patients with BIPI. *P* < 0.05 was considered significant. Conditional logistic regression analysis was performed to evaluate the association between urinary 6-SMT levels and BIPI.

## Results

### Comparison of general information between the two groups

There were 30 very preterm infants with brain injury, of whom 16 were male and 14 were female. In the control group, there were 97 subjects, including 49 males and 48 females. There were no statistical differences with regards to gender, mode of delivery, asphyxia, and exposure to ACS between the two groups (*p* > 0.05). The brain injury group had significantly higher rates of early infection and asphyxia than the control group (*P* < 0.05). The brain injury group exhibited a significantly lower incidence of MgSO4 exposure, acidosis, and lower urinary 6-SMT concentrations on days 1, 3, and 7 compared to the control group (all *P* < 0.05), as shown in Table 1.

**Table 1:** Comparison of baseline data and urinary 6-SMT levels between case group and control group.

Characteristics	Brain Injury Group (*n* = 30)	Control Group (*n* = 97)	(Z/χ²)	*p* value
GA (weeks)	29.36(28.40-30.61)	30.43(29.15–31.29)	– 2.20	0.028*
BW (g)	1.21(1.03–1.44)	1.34(1.13–1.59)	– 2.17	0.030*
Gender				
Male	16(53.3%)	49(50.5%)	0.07	0.787
Female	14(46.7%)	48(49.5%)		
Delivery mode				
Vaginal	14(46.7%)	58(59.8%)	0.63	0.531
Cesarean	16(53.3%)	39(40.2%)		
Acidosis				
Yes	3(10.0%)	12(12.4%)	5.16	0.023*
No	27(90.0%)	85(87.6%)		
Asphyxia				
Yes	9(30.0%)	1(1.0%)	6.04	0.014*
No	21(70.0%)	96(99.0%)		
ACS				
Yes	26(86.7%)	93(95.9%)	1.92	0.166
No	4(13.3%)	4(4.1%)		
MgSO₄				
Yes	9(30.0%)	59(60.8%)	8.75	0.003*
No	21(70.0%)	38(39.2%)		
Early infection				
Yes	14(46.7%)	21(21.6%)	7.18	0.007*
No	16(53.3%)	76(78.4%)		
UM1 (pg/mL)	558.51(430.03-776.74)	813.86(432.59-1329.41)	-2.42	0.015*
UM2 (pg/mL)	722.62(583.48–808.20)	938.48(739.66-1187.55)	-3.54	<0.001*
UM3 (pg/mL)	796.81(650.02-918.09)	1034.48(735.74-1462.90)	-2.46	0.014*

* indicates statistical significance (*p* < 0.05). Nonnormal variables were reported as median (inter-quartile range), and binary variables were represented as the proportions. Compare the parameters between two groups of patients using the non-parametricor chi-square tests. UM1, urinary 6-SMT on day 1; UM2, urinary 6-SMT on day 3; UM3, urinary 6-SMT on day 7.

When the 30 brain injury infants were stratified into mild-moderate (*n* = 26) versus severe (*n* = 4) injury, urinary 6-SMT levels were slightly higher in the severe group at all time-points, yet none of these differences reached statistical significance (Supplementary Table 2). The absence of a significant gradient is most likely explained by the very small number of severe cases.

### ROC analysis

**Fig. 1:**
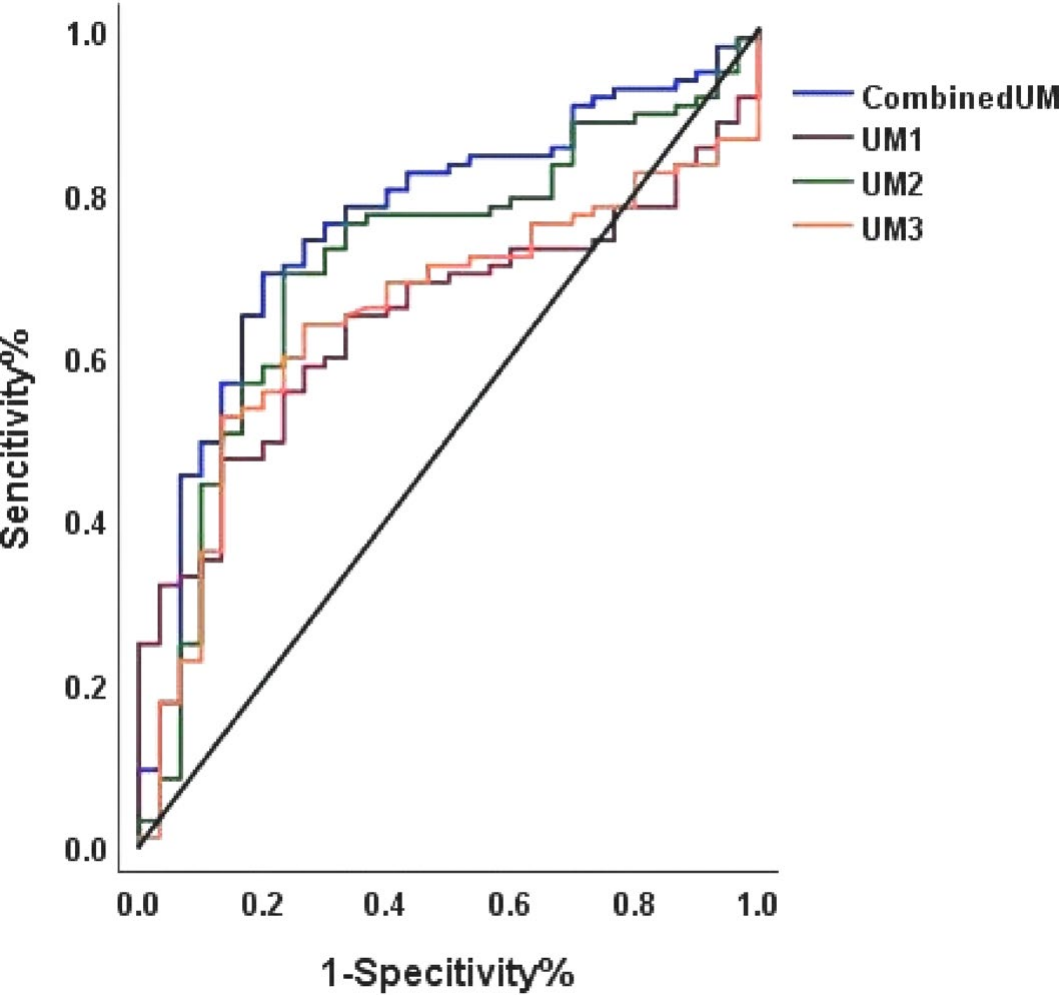
ROC curves of urinary 6-SMT for diagnosing BIPI in very preterm infants. UM1: Urinary 6-SMT on day 1; UM2: Urinary 6-SMT on day 3; UM3: Urinary 6-SMT on day 7; CombinedUM: Integrated model of 6-SMT using days 1, 3 and 7; Black line: Reference line for random chance (AUC = 0.5).

Urinary 6-SMT levels at different postnatal time points showed varying discriminative ability for identifying BIPI in very preterm infants. The AUC values were 0.647 (95% CI: 0.551–0.742; *p* = 0.003) on day 1, 0.714 (95% CI: 0.611–0.817; *p* < 0.001) on day 3, and 0.649 (95% CI: 0.547–0.751; *p* = 0.004) on day 7. A combined model integrating 6-SMT levels across all three time points achieved the highest discriminative power, with an AUC of 0.764 (95% CI: 0.669–0.858, *p* < 0.001). At the optimal threshold 708.44, this model provided a sensitivity of 78.4% and specificity of 66.7%, outperforming individual time-point measurements. (Table 2; Fig. 1).

**Table 2:** Diagnostic value of urinary 6-SMT in patients with BIPI.

Index	AUC	Cut-off value	Sensitivity	Specificity	95% CI	*P*
UM1	0.647	597.00	69.1%	56.7%	0.551–0.742	0.003*
UM2	0.714	762.46	73.2%	70.0%	0.611–0.817	<0.001*
UM3	0.649	843.98	69.1%	60.0%	0.547–0.751	0.004*
CombinedUM	0.764	708.44	78.4%	66.7%	0.669–0.858	<0.001*

* indicates statistical significance (*p* < 0.05). UM1, Urinary 6-SMT on Day 1; UM2, Urinary 6-SMT on Day 3; UM3, Urinary 6-SMT on Day 7; CombinedUM, Integrated model of 6-SMT using days 1, 3 and 7.

### Longitudinal trends of urinary 6-SMT and their relationship with other clinical indicators

**Fig. 2:**
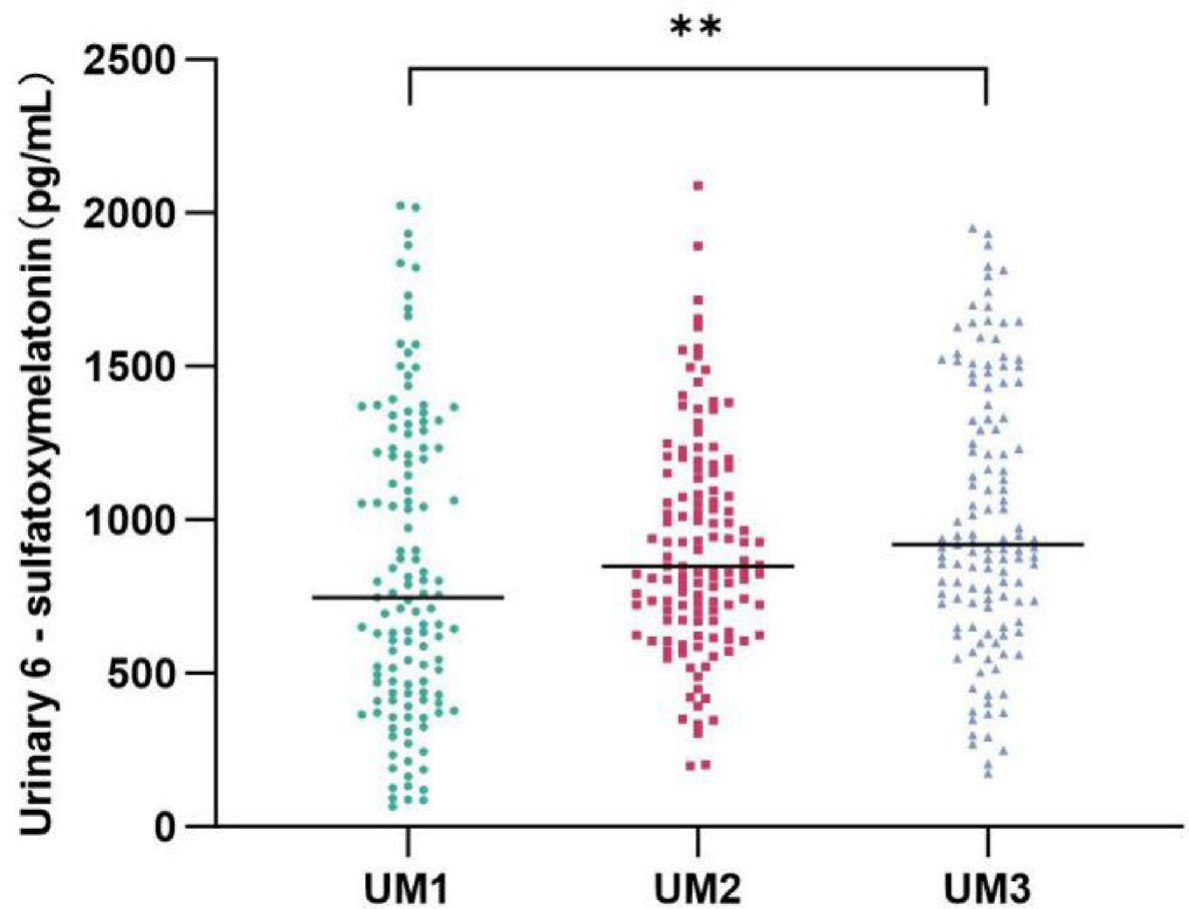
Urinary 6-SMT levels across postnatal days 1, 3, and 7. UM1, urinary 6-SMT on day 1; UM2, urinary 6-SMT on day 3; UM3, urinary 6-SMT on day 7.

Comparative analysis of urinary 6-SMT concentrations in very preterm infants revealed significant differences across postnatal days 1, 3, and 7 (*P* < 0.01). The levels demonstrated a progressive increase with advancing postnatal age, as presented in Table 3; Fig. 2.

**Table 3:** Urinary 6-SMT levels by postnatal day in very preterm infants.

Time	Urinary 6-SMT (pg/mL)	H	*P*
Day 1	747.23(434.61-1,233.70)	10.79	0.005*
Day 3	847.98(673.12-1,151.49)		
Day 7	919.48(715.63-1,374.32)		

* indicates statistical significance (*p* < 0.05). variables were reported as median (inter-quartile range). Group comparisons were performed using Kruskal-Wallis test.

**Fig. 3:**
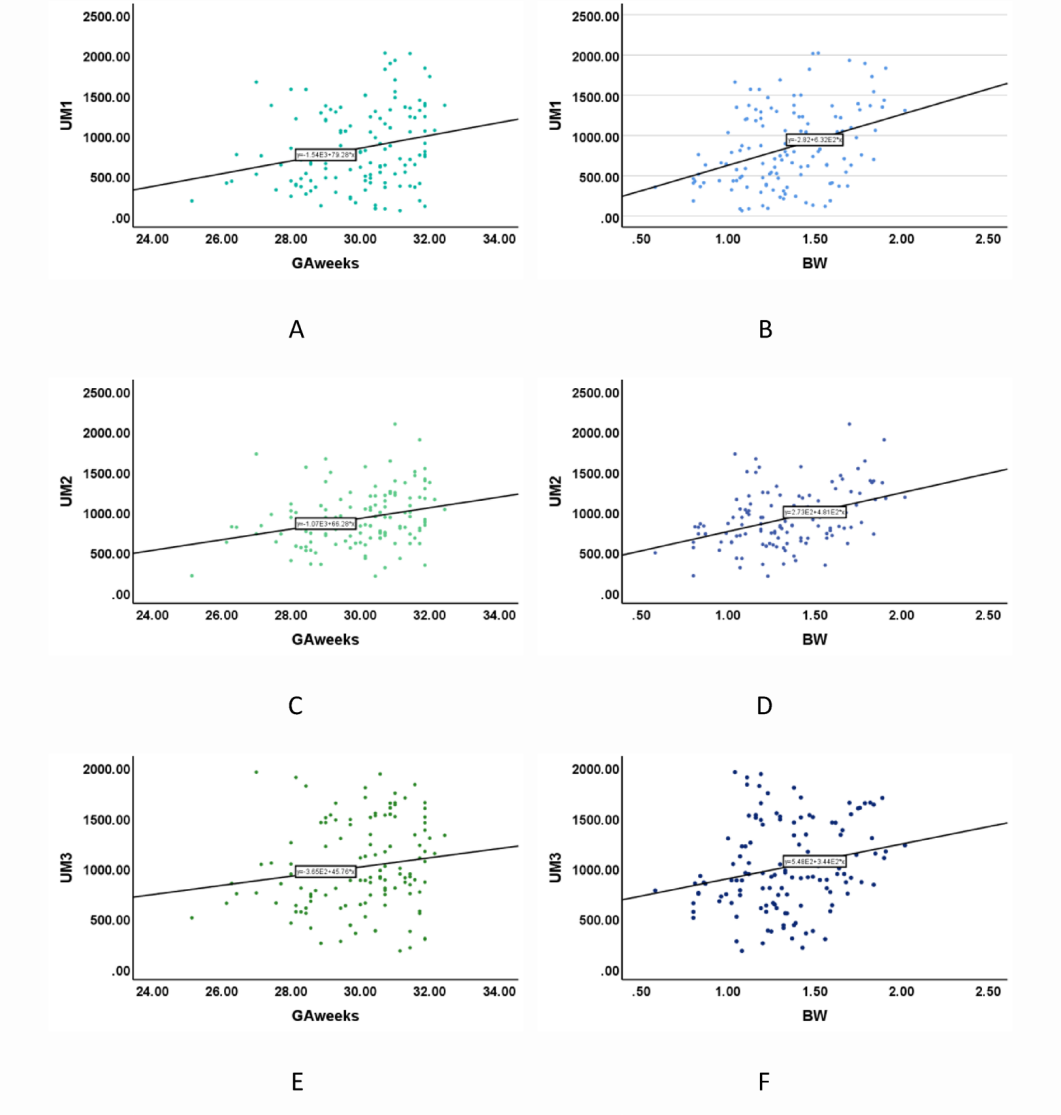
Correlations of urinary 6-SMT levels with GA and BW in very preterm infants.

Urinary 6-SMT levels showed significant positive correlations with GA and BW in very preterm infants during the first 3 postnatal days. Specifically, low-to-moderate correlations were observed on day 1 (GA: *r* = 0.25, *p* = 0.004; BW: *r* = 0.34, *p* < 0.001) and day 3 (GA: *r* = 0.31, *p* < 0.001; BW: *r* = 0.36, *p* < 0.001). However, by Day 7, the correlations diminished to negligible strengths (GA: *r* = 0.04, *p* = 0.030; BW: *r* = 0.01, *p* = 0.008), indicating minimal practical relevance despite statistical significance (Table 4; Fig. 3). Antenatal MgSO₄ exposure was associated with significantly lower urinary 6-SMT levels on Day 1 (Z = -3.03, *p* = 0.002) and Day 3 (Z = -2.13, *p* = 0.033), though this difference was no longer apparent by Day 7 (Z = -7.49, *p* = 0.454). No significant associations were found between urinary 6-SMT levels and ACS, neonatal acidosis, asphyxia, early infection, or infant sex at any time point(*p* > 0.05 for all comparisons). (Table 4).

**Table 4:** Urinary 6-SMT levels and perinatal correlates in very preterm infants.

	UM1		UM2		UM3	
	*r*/Z	*P*	*r*/Z	*P*	*r*/Z	*P*
GA	0.25	0.004*	0.31	< 0.001*	0.04	0.030*
BW	0.34	< 0.001*	0.36	< 0.001*	0.01	0.008*
MgSO₄	– 3.03	0.002*	– 2.13	0.033*	– 7.49	0.454
Gender	– 0.62	0.531	– 1.03	0.302	– 0.88	0.377
ACS	– 1.42	0.153	– 1.66	0.095	– 1.08	0.279
Acidosis	– 0.18	0.851	– 0.28	0.775	– 1.40	0.161
Asphyxia	– 0.89	0.370	– 1.62	0.103	– 3.17	0.751
Early infection	– 0.79	0.425	– 0.66	0.503	– 0.10	0.920

* indicates statistical significance (*p* < 0.05). Statistical analyses were performed using the Mann-Whitney U test for categorical variables (e.g., MgSO_4_, gender) and Spearman’s correlation for continuous variables (e.g., GA, BW). Significant results are shown in bold.

UM1, urinary 6-SMT on day 1; UM2, urinary 6-SMT on day 3; UM3, urinary 6-SMT on day 7.

Scatter plots showing Spearman’s correlations between 6-SMT levels and GA (A, C, E) or BW (B, D, F) on postnatal days 1, 3, and 7 in very preterm infants. Solid lines represent the Loess fit. UM1, urinary 6-SMT on day 1; UM2, urinary 6-SMT on day 3; UM3, urinary 6-SMT on day 7. 6-SMT (pg/mL), GA (weeks), BW (kg).

### Matching and conditional logistic regression analysis

After identifying GA and BW as the variables most strongly correlated with 6-SMT (Table 4), we used these two factors for 1:1 nearest-neighbor matching. Each case was paired with the most comparable control (Supplementary Table 1).

To evaluate the association between urinary melatonin metabolites and brain injury in very preterm infants, we performed conditional logistic regression analysis. First, a base model containing only urinary metabolites showed a significant protective effect of UM2 (OR = 0.997, *p* = 0.005). UM1 yielded a statistically significant but biologically negligible risk increase (OR = 1.002, 95% CI 1.000–1.004, *p* = 0.049). Subsequently, an adjusted model incorporated gestational age difference (ΔGA) and birth weight difference (ΔBW) to control for confounding effects. After adjustment, the protective effect of UM2 strengthened (OR = 0.996, *p* = 0.004), whereas the negligible effect of UM1 was no longer statistically significant (OR = 1.002, 95% CI 1.000–1.004, *p* = 0.070). Model fit significantly improved (likelihood ratio test: χ² = 6.7, df = 2, *p* = 0.035). Complete results are presented in Table 5.

**Table 5:** Conditional logistic regression analysis of urinary metabolites.

Variable	Base model		Adjusted model	
	OR (95% CI)	*p*-value	OR (95% CI)	*p*-value
UM1	1.002 (1.000-1.004)	0.049*	1.002 (1.000-1.004)	0.070
UM2	0.997 (0.994–0.999)	0.005*	0.996 (0.994–0.999)	0.004*
UM3	1.001 (0.999–1.003)	0.271	1.001 (0.999–1.003)	0.258

* indicates statistical significance (*p* < 0.05). The adjusted model controlled for ΔGA and ΔBW between matched pairs. ΔGA: OR = 1.422 (95% CI: 0.995–2.032), *p* = 0.054; ΔBW: OR = 0.151 (95% CI: 0.027–0.839), *p* = 0.030. Model fit improved significantly after adjustment (Likelihood ratio test: χ² = 6.7, df = 2, *p* = 0.035).

## Discussion

Preterm infants are exposed to multiple prenatal adversities and postnatal invasive procedures in the neonatal intensive care unit, which collectively contribute to elevated oxidative stress levels. Melatonin levels can vary significantly due to factors that increase oxidative stress in preterm infants. Our study revealed that infants with BIPI had significantly lower urinary 6-SMT concentrations on days 1, 3 and 7, together with a higher incidence of early infection and asphyxia, and lower antenatal MgSO₄ exposure and acidosis than the control group (*P* < 0.05). Moreover, the levels of urinary 6-SMT exhibited a progressive increase during the first week of life, positively correlating with advancing postnatal age (Days 1–7). This trend is consistent with the physiological recovery and maturation of the melatonin production system in very preterm infants. Furthermore, 6-SMT levels demonstrated significant associations with GA, BW, and antenatal MgSO₄ exposure, which are important clinical indicators in the management of preterm infants. Notably, our findings suggest that urinary 6-SMT has substantial potential diagnostic utility for identifying BIPI in very preterm infants. Both the ROC analysis and the conditional logistic regression analysis highlighted the significant predictive value of urinary 6-SMT levels, with the combined model data indicating a strong association with brain injury. These results collectively highlight the potential of urinary 6-SMT as a biomarker for early prediction of BIPI in very preterm infants, which may aid in timely intervention and improved clinical outcomes.

Infants exposed to antenatal MgSO₄ exhibited higher 6-SMT levels and a lower incidence of brain injury, consistent with large trials showing MgSO₄ reduces cerebral palsy via NMDA-receptor blockade and antioxidant potentiation^13^. This is consistent with randomized trials and meta-analyses showing antenatal MgSO₄ lowers cerebral palsy and brain injury rates^14^.

Our findings demonstrate significantly lower urinary 6-SMT levels in very preterm infants with BIPI compared to unaffected controls, suggesting a potential role of melatonin deficiency in the pathophysiology of preterm brain injury. This inverse association aligns with recent clinical evidence from Cheng et al.^15^, who identified serum melatonin as an independent risk factor for brain injury in preterm infants. Notably, their reported serum melatonin levels in brain-injured infants fall within the range of urinary 6-SMT concentrations observed in our cohort, supporting the translational relevance of urinary 6-SMT as a noninvasive biomarker. Furthermore, we observed a progressive increase in urinary 6-SMT concentrations during the first postnatal week, a developmental pattern not previously characterized in very preterm populations. Notably, current literature reports only one study by Biran^16^ et al. involving 110 preterm infants < 34 weeks of gestation, which documented cross-sectional measurements of urinary 6-SMT levels at unspecified postnatal time points. Critically, their study did not analyze dynamic changes in 6-SMT concentrations across specific postnatal days. In contrast, our study systematically characterizes, for the first time, a distinct postnatal age-dependent increase in urinary 6-SMT levels among very preterm infants during the first week of life. This trajectory, marked by progressive elevation from Day 1 to Day 7, addresses a critical gap in understanding melatonin metabolism in the most vulnerable preterm population and provides foundational data for investigating the temporal relationship between melatonin ontogeny and neuroprotective repair mechanisms in extreme prematurity. Experimental studies substantiate the neuroprotective potential of melatonin in brain injury models. Garofoli^17^ et al. (GA ≤ 29 + 6 weeks) demonstrated oral melatonin’s antioxidant efficacy and neuroprotective pharmacokinetics in preterm newborns, while Hakiminia et al.^18^ reported mitochondrial protection via melatonin administration in acquired brain injuries. Mechanistically, Liu^19^ et al. identified PI3K/AKT/Nrf2 pathway activation as a key mediator of melatonin’s anti-edema effects. Our observations of diminished 6-SMT levels in infants with BIPI and their postnatal developmental trajectory suggest that endogenous melatonin depletion may exacerbate oxidative stress and impair neuroprotective responses in vulnerable preterm brains.

To evaluate the diagnostic potential of urinary 6-SMT for BIPI, we performed ROC analysis. The derived AUC values demonstrated that diminished 6-SMT levels may serve as a diagnostic biomarker associated with BIPI, with the highest discriminative capacity observed in the combined model integrating serial 6-SMT measurements across postnatal days 1–7. Notably, this multi-timepoint model exhibited superior diagnostic accuracy compared with single-day 6-SMT assessments, suggesting dynamic monitoring enhances predictive utility. Our findings align with Cheng et al.^15^, who identified serum melatonin as a risk predictor for brain injury across gestational ages. Parallel evidence from adult populations further supports melatonin’s biomarker potential: Hou et al.^20^ reported altered serum melatonin levels in hypertensive intracerebral hemorrhage, though their focus on blood-based markers differs from our urinary 6-SMT approach. Critically, to our knowledge, this is the first study to systematically characterize urinary 6-SMT as a noninvasive diagnostic biomarker for BIPI in very preterm infants. Unlike invasive blood sampling, urinary 6-SMT quantification offers practical advantages for serial monitoring in fragile neonates. Future studies should validate these findings in multicenter cohorts and explore optimal sampling timepoints to maximize clinical translation.

Our analysis further identified significant associations between urinary 6-SMT levels and GA, BW, and antenatal MgSO₄exposure. Using GA and BW as matching variables, we performed 1:1 nearest-neighbor matching and conducted pre- and post-matching conditional logistic regression analyses. These analyses revealed a more pronounced protective effect for day 3 6-SMT levels after correcting for GA and BW discrepancies. Gestational age exhibited a weak positive correlation with 6-SMT concentrations on postnatal day 1, which strengthened to a moderate correlation by day 3 before diminishing to a negligible association by day 7. This dynamic pattern aligns with Sánchez-Borja et al.^21^, who reported reduced melatonin levels in preterm infants < 32 weeks, suggesting developmental immaturity of melatonin synthesis pathways in early gestation. Similarly, birth weight demonstrated moderate positive correlations with 6-SMT levels on days 1 and 3, transitioning to a minimal correlation by day 7. These findings contrast with Pavlyshyn^22^ et al., who observed no significant BW-dependent melatonin variations in their preterm cohort, potentially reflecting differences in sampling protocols or population characteristics. Notably, neonates exposed to antenatal MgSO₄ exhibited elevated 6-SMT concentrations on days 1 and 3 compared to unexposed infants. We speculate that MgSO₄-mediated neuroprotection lowers acute oxidative stress, thereby transiently down-regulating endogenous melatonin consumption and leaving more of its metabolite to be excreted. This inverse association does not contradict the neuroprotective synergy observed in animal models but reflects a short-term metabolic adaptation that warrants longitudinal validation. Experimental studies provide mechanistic insights: MgSO₄ and melatonin synergistically mitigate hypoxic-ischemic brain injury in rodent models by reducing cerebral infarct volume and apoptosis^23^. As a fetal neuroprotectant, MgSO_4_ attenuates excitotoxicity via NMDA receptor blockade, modulating calcium influx and glutamatergic signaling^24^. Our study found no significant association between urinary 6-SMT levels and early infections. This contrasts with preclinical evidence that chorioamnionitis is a key placental inflammatory stressor, which disrupts melatonin homeostasis through the placental-fetal-brain axis, as demonstrated by Kitaseet al.^25^, who observed suppressed melatonin signaling in inflammation-driven models. The divergence between our clinical observations and preclinical chorioamnionitis data may reflect differences in infection timing (antenatal vs. postnatal), pathogen-specific effects, or compensatory mechanisms in human neonates. These observations collectively underscore the complex interplay between perinatal stressors, neuroprotective interventions, and melatonin dynamics in preterm neonates.

This study has several limitations to acknowledge. First, the lack of healthy full-term controls prevents direct comparisons of urinary 6-SMT levels between preterm and term populations, limiting our understanding of developmental baselines. Second, the relatively small sample size may affect generalizability, necessitating validation in larger, multicenter cohorts. While preclinical studies support melatonin’s neuroprotective potential in brain injury models (e.g., hypoxic-ischemic and inflammation-driven mechanisms), our findings highlight the need for mechanistic research to clarify how GA and other factor-dependent melatonin synthesis modulates oxidative stress in BIPI. Future research should therefore combine longitudinal tracking of 6-SMT trajectories with neurodevelopmental follow-up, and test melatonin supplementation guided by serial 6-SMT monitoring. With an assay cost of only USD 1.7 per sample, such serial testing is economically feasible even in resource-limited settings and may facilitate the translation of 6-SMT from a diagnostic biomarker to a theranostic tool in neonatal care.

## Conclusion

This study demonstrates that very preterm infants with brain injury exhibit significantly lower urinary 6-SMT levels compared with controls across the first postnatal week. The combined model integrating urinary 6-SMT levels across multiple time points shows superior diagnostic potential for brain injury in preterm neonates compared with single-time-point assessments. The progressive increase in 6-SMT levels during early postnatal life and their significant associations with gestational age and birth weight suggest developmental maturation of melatonin synthesis pathways. Additionally, antenatal MgSO₄ exposure is associated with lower urinary 6-SMT levels on days 1 and 3, indicating potential interactions between melatonin metabolism and perinatal interventions. These findings indicate that melatonin deficiency may contribute to preterm brain injury pathophysiology and support further exploration of urinary 6-SMT as a noninvasive biomarker for clinical validation.

## Supplementary Information

Below is the link to the electronic supplementary material.

Supplementary Material 1

Supplementary Material 2

Supplementary Material 3

## Data Availability

The datasets generated and/or analyzed during the current study are available in the OSF repository, https://doi.org/10.17605/OSF.IO/AWQ49.
