# Supplementary material for: Urinary 6-sulfatoxymelatonin as a predictive biomarker for brain injury in very preterm infants
Source: Sci Rep. 2026 Feb 27;16:11254. doi: 10.1038/s41598-026-42005-0 (PMC13049170; doi:10.1038/s41598-026-42005-0)
Supplement: Supplementary file 1 — Supplementary Material 1 [file 41598_2026_42005_MOESM1_ESM.docx]

Supplementary Table 1 Matching Results between brain injury group and control group

| Pair ID | Group | UM1 (pg/mL) | UM2 (pg/mL) | UM3 (pg/mL) | GA (weeks) | BW (kg) |
| --- | --- | --- | --- | --- | --- | --- |
| 1 | 1 | 589.34 | 723.77 | 949.93 | 29.00 | 1.10 |
|  | 0 | 1198.70 | 1023.95 | 1212.66 | 29.00 | 1.10 |
| 2 | 1 | 898.18 | 992.27 | 973.48 | 30.57 | 1.33 |
|  | 0 | 618.91 | 669.91 | 905.87 | 30.57 | 1.37 |
| 3 | 1 | 437.07 | 521.39 | 667.23 | 28.57 | 0.95 |
|  | 0 | 658.91 | 623.08 | 715.63 | 28.57 | 0.96 |
| 4 | 1 | 517.79 | 724.79 | 750.79 | 27.00 | 0.83 |
|  | 0 | 762.37 | 805.99 | 742.98 | 26.43 | 0.83 |
| 5 | 1 | 542.13 | 735.69 | 911.32 | 30.57 | 1.25 |
|  | 0 | 608.24 | 674.02 | 896.87 | 30.57 | 1.42 |
| 6 | 1 | 634.12 | 724.12 | 842.12 | 27.71 | 0.87 |
|  | 0 | 325.23 | 624.58 | 650.84 | 27.57 | 1.02 |
| 7 | 1 | 638.32 | 671.88 | 881.63 | 31.57 | 1.57 |
|  | 0 | 1219.79 | 1448.55 | 1328.79 | 31.57 | 1.65 |
| 8 | 1 | 758.94 | 866.78 | 857.82 | 31.86 | 1.77 |
|  | 0 | 1369.94 | 1386.29 | 1647.96 | 31.86 | 1.82 |
| 9 | 1 | 574.89 | 698.47 | 874.93 | 29.71 | 1.05 |
|  | 0 | 1118.18 | 1238.92 | 1429.45 | 29.71 | 1.20 |
| 10 | 1 | 1232.29 | 1193.67 | 1231.56 | 30.29 | 1.41 |
|  | 0 | 512.16 | 1019.23 | 1501.57 | 30.29 | 1.42 |
| 11 | 1 | 469.76 | 673.12 | 729.28 | 30.29 | 1.29 |
|  | 0 | 659.68 | 827.34 | 904.39 | 30.29 | 1.37 |
| 12 | 1 | 187.39 | 203.79 | 503.46 | 25.14 | 0.80 |
|  | 0 | 430.58 | 812.72 | 845.85 | 26.29 | 0.81 |
| 13 | 1 | 408.79 | 621.02 | 648.37 | 26.14 | 0.80 |
|  | 0 | 747.23 | 827.59 | 1034.48 | 27.14 | 1.30 |
| 14 | 1 | 244.46 | 393.59 | 450.72 | 28.00 | 1.39 |
|  | 0 | 842.02 | 1010.50 | 937.38 | 28.00 | 1.12 |
| 15 | 1 | 1323.12 | 1487.87 | 1524.37 | 29.14 | 1.16 |
|  | 0 | 650.18 | 927.88 | 948.56 | 29.00 | 1.20 |
| 16 | 1 | 322.38 | 586.79 | 650.57 | 30.14 | 1.26 |
|  | 0 | 437.84 | 421.39 | 513.96 | 30.14 | 1.05 |
| 17 | 1 | 403.12 | 794.91 | 759.12 | 31.71 | 1.59 |
|  | 0 | 190.49 | 765.73 | 569.38 | 31.71 | 1.59 |
| 18 | 1 | 463.46 | 554.39 | 562.48 | 28.29 | 0.80 |
|  | 0 | 365.00 | 848.91 | 919.48 | 28.29 | 0.84 |
| 19 | 1 | 473.44 | 704.85 | 728.46 | 29.00 | 1.07 |
|  | 0 | 1368.53 | 1372.71 | 1449.47 | 29.00 | 1.60 |
| 20 | 1 | 357.79 | 489.35 | 774.74 | 28.71 | 0.58 |
|  | 0 | 126.08 | 346.80 | 249.30 | 28.86 | 1.20 |
| 21 | 1 | 527.46 | 564.96 | 599.53 | 28.43 | 1.28 |
|  | 0 | 710.82 | 754.48 | 929.08 | 28.43 | 1.27 |
| 22 | 1 | 392.46 | 573.57 | 563.43 | 28.14 | 1.22 |
|  | 0 | 379.16 | 572.23 | 624.84 | 28.14 | 1.22 |
| 23 | 1 | 630.79 | 729.03 | 797.38 | 30.71 | 1.57 |
|  | 0 | 412.35 | 704.83 | 627.40 | 30.71 | 1.60 |
| 24 | 1 | 830.12 | 931.51 | 879.36 | 30.71 | 1.53 |
|  | 0 | 1821.70 | 1203.35 | 1522.92 | 30.71 | 1.47 |
| 25 | 1 | 873.12 | 847.98 | 598.96 | 29.43 | 1.19 |
|  | 0 | 1034.82 | 853.84 | 1292.98 | 29.43 | 1.00 |
| 26 | 1 | 701.46 | 721.47 | 831.73 | 31.43 | 1.84 |
|  | 0 | 1044.19 | 996.37 | 938.57 | 31.43 | 1.63 |
| 27 | 1 | 474.79 | 593.68 | 796.25 | 29.29 | 1.20 |
|  | 0 | 294.16 | 927.61 | 1475.85 | 29.29 | 1.19 |
| 28 | 1 | 1184.12 | 1656.02 | 1498.29 | 29.00 | 1.16 |
|  | 0 | 604.66 | 735.98 | 634.91 | 30.00 | 1.27 |
| 29 | 1 | 1210.77 | 759.20 | 1931.79 | 30.57 | 1.19 |
|  | 0 | 66.38 | 418.57 | 174.28 | 31.14 | 1.08 |
| 30 | 1 | 711.63 | 794.94 | 938.42 | 31.14 | 1.67 |
|  | 0 | 371.08 | 818.39 | 995.38 | 31.14 | 1.64 |

Note: UM1, Urinary 6-SMT on day 1; UM2, Urinary 6-SMT on day 3; UM3, Urinary 6-SMT on day 7.
