# Supplementary material for: Urinary 6-sulfatoxymelatonin as a predictive biomarker for brain injury in very preterm infants
Source: Sci Rep. 2026 Feb 27;16:11254. doi: 10.1038/s41598-026-42005-0 (PMC13049170; doi:10.1038/s41598-026-42005-0)
Supplement: Supplementary file 2 — Supplementary Material 2 [file 41598_2026_42005_MOESM2_ESM.docx]

Supplementary Table 2. Urinary 6-SMT levels according to severity of brain injury in 30 very preterm infants

| Index | Mild-moderateinjury (n = 26)  Median (IQR), pg/mL | Severe injury (n = 4)  Median (IQR), pg/mL | Z | p |
| --- | --- | --- | --- | --- |
| UM1 | 558.5 (407.4 – 776.7) | 609.6 (484.5 – 1100) | -0.73 | 0.46 |
| UM2 | 711.1 (571.4 – 808.2) | 723.1 (709.0 – 1076) | -0.79 | 0.43 |
| UM3 | 796.8 (636.2 – 918.1) | 791.3 (734.0 – 1132) | -0.24 | 0.81 |

Note: Mild-moderate injury: Papile grade 1-2 or persistent punctate white-matter lesions. Severe injury: Papile grade 3–4 or cystic periventricular leukomalacia.
