# Supplementary material for: Urinary 6-sulfatoxymelatonin as a predictive biomarker for brain injury in very preterm infants
Source: Sci Rep. 2026 Feb 27;16:11254. doi: 10.1038/s41598-026-42005-0 (PMC13049170; doi:10.1038/s41598-026-42005-0)
Supplement: Supplementary file 3 — Supplementary Material 3 [file 41598_2026_42005_MOESM3_ESM.docx]

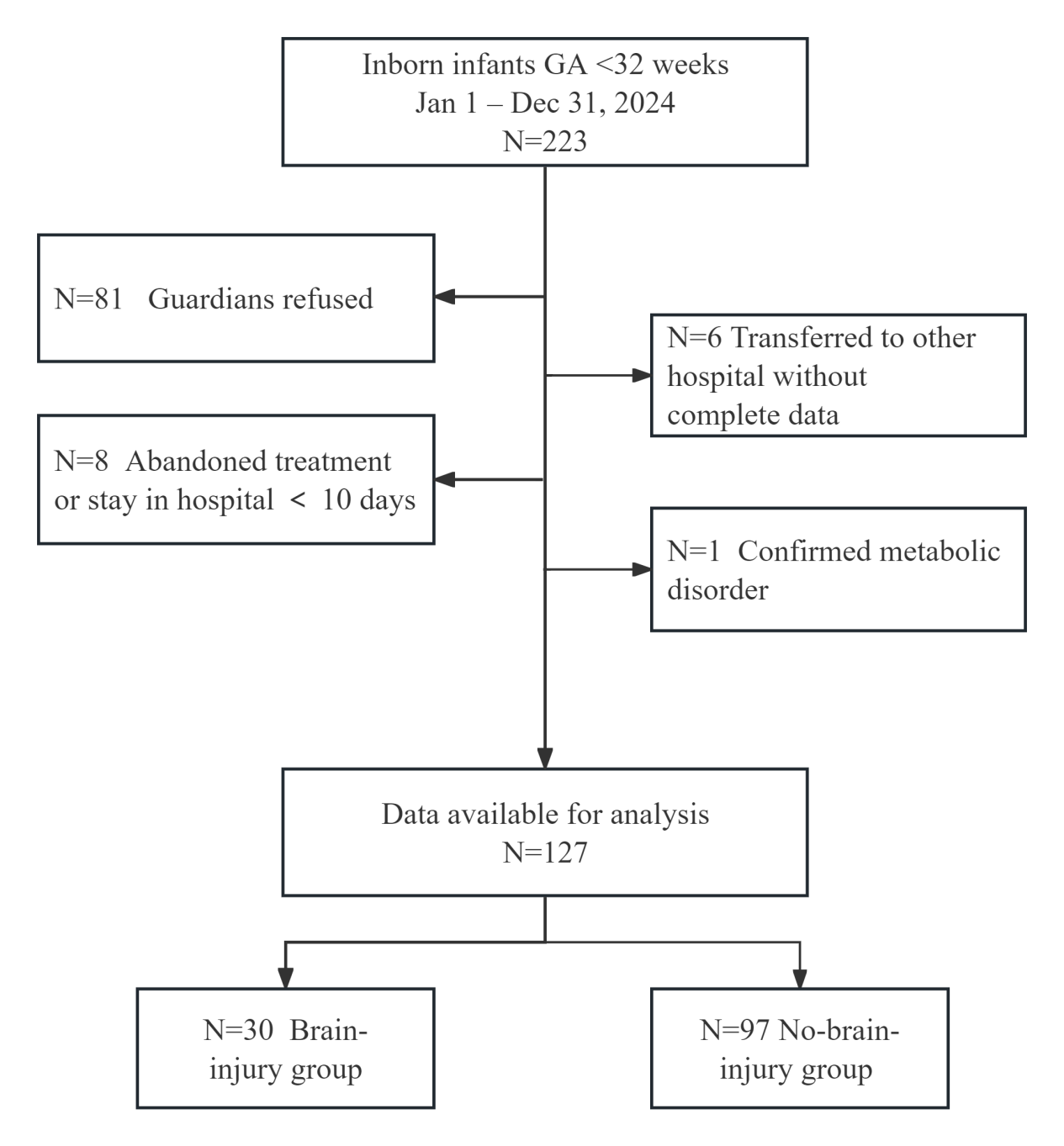


Fig. S1. Patient flow chart

Of 223 screened infants, 96 were excluded for various reasons, leaving 127 enrolled and classified into brain-injury (n=30) or no-brain-injury (n=97) groups based on imaging findings.
